# Supplementary material for: Humoral Responses against BQ.1.1 Elicited after Breakthrough Infection and SARS-CoV-2 mRNA Vaccination
Source: Vaccines (Basel). 2023 Jan 21;11(2):242. doi: 10.3390/vaccines11020242 (PMC9963157; doi:10.3390/vaccines11020242)
Supplement: Supplementary file 1 [file vaccines-11-00242-s001.zip › vaccines-2141799-supplementary.pdf]

Supplementary Material

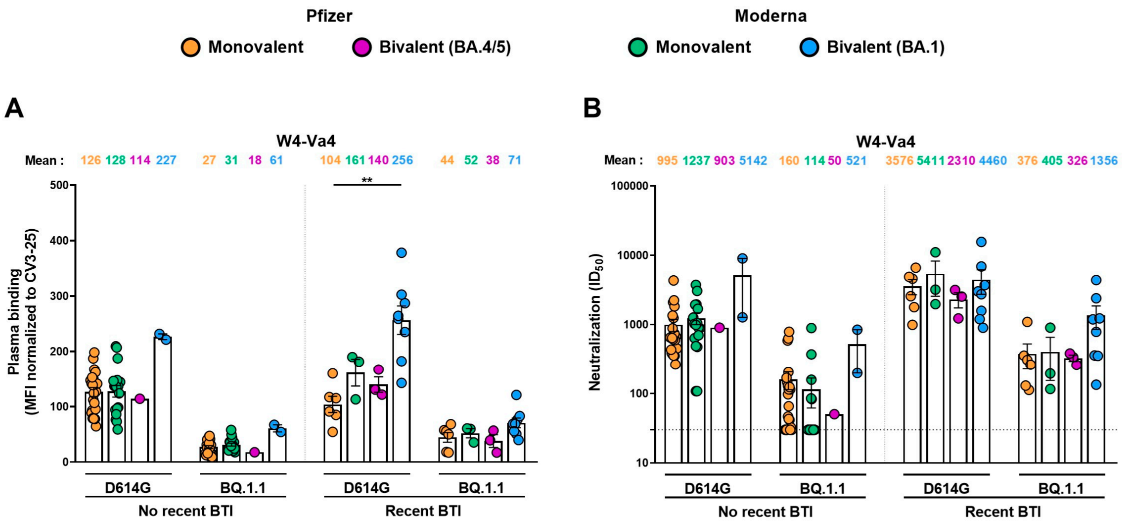

Figure S1. Recognition and neutralization of the D614G and BQ.1.1 Spikes after the fourth doses of SARS-CoV-2 vaccine in individuals with or without a recent breakthrough infection. (A) 293T cells were transfected with the full-length D614G or BQ.1.1 S, stained with the CV3-25 mAb or with plasma from vaccinated individuals and analyzed by flow cytometry. The values represent the MFI normalized by CV3-25 mAb binding. (B) Neutralization activity was measured by incubating pseudoviruses bearing SARS-CoV-2 S glycoproteins, with serial dilutions of plasma for 1 h at 37°C before infecting 293T-ACE2 cells. Neutralization half maximal inhibitory serum dilution (ID_{50}) values were determined using a normalized non-linear regression using GraphPad Prism software. Individuals vaccinated with Pfizer monovalent, Moderna monovalent, Pfizer bivalent (BA.4/5) or Moderna bivalent (BA.1) fourth dose are represented by orange, green, purple and blue points respectively. Limits of detection are plotted. Error bars indicate means ± SEM. (** $P < 0.01$).
